# Circumvallate Placenta: Associated Clinical Manifestations and Complications—A Retrospective Study

**DOI:** 10.1155/2014/986230

**Published:** 2014-11-13

**Authors:** Hanako Taniguchi, Shigeru Aoki, Kentaro Sakamaki, Kentaro Kurasawa, Mika Okuda, Tsuneo Takahashi, Fumiki Hirahara

**Affiliations:** ^1^Perinatal Center for Maternity and Neonate, Yokohama City University Medical Center, 4-57 Urafunecyou, Minami-ku, Yokohama, Kanagawa 232-0024, Japan; ^2^Department of Biostatistics and Epidemiology, Yokohama City University Graduate School of Medicine and University Medical Center, Yokohama, Japan; ^3^Department of Obstetrics and Gynecology, Yokohama City University Hospital, Yokohama, Japan

## Abstract

*Aims.* To analyze the pregnancy outcomes of circumvallate placenta retrospectively and to predict circumvallate placenta during pregnancy based on its clinical features. *Methods.* The pregnancy outcomes of 92 women with circumvallate placenta who delivered live singletons at a tertiary care center between January 2000 and September 2012 were compared with those of 9057 controls. *Results.* Women with circumvallate placenta were associated with higher incidences of preterm delivery (64.1%), placental abruption (10.9%), emergency cesarean section (45.6%), small-for-gestational age (36.9%), neonatal death (8.9%), neonatal intensive care unit admission (55.4%), and chronic lung disease (33.9%). When vaginal bleeding during the second trimester and premature chemical rupture of membranes (PCROM) were both used as predictive factors for circumvallate placenta, the sensitivity was 28.8% and specificity was 99.9%. *Conclusion.* With circumvallate placenta, pregnancy outcomes were poor and had characteristic clinical manifestations. In women with both vaginal bleeding and PCROM during pregnancy, circumvallate placenta should be strongly suspected.

## 1. Introduction

Circumvallate placenta is a form of extrachorial placenta, with a raised placental margin in an annular shape. The chorionic plate is smaller than the basal plate, and misalignment between them causes hematoma retention in the placental margin. Thus, circumvallate placenta often causes persistent vaginal bleeding beginning in the 1st trimester, premature rupture of the membranes (PROM), preterm delivery, and placental abruption and is thus associated with poor pregnancy outcomes [1]. 

However, few circumvallate placenta cases have been reported. The diagnosis is difficult during pregnancy and circumvallate placenta is often detected only by placental examination after delivery [2–5].

Herein, we reviewed retrospectively the pregnancy outcomes in women with circumvallate placenta. We also evaluated the usefulness of using clinical features including premature chemical rupture of membranes (PCROM) and vaginal bleeding in predicting the occurrence of circumvallate placenta prenatally.

## 2. Materials and Methods

We retrospectively analyzed medical records of women who delivered live singletons after 22 gestational weeks and were diagnosed with completely circumvallate placenta by postdelivery placental macroscopic examination at the Perinatal Center for Maternity and Neonate of Yokohama City University Medical Center between January 2000 and September 2012. Women with fetal abnormalities, uterine abnormalities, cervical polyps that might cause vaginal bleeding, placenta previa, and inherited bleeding diathesis including thrombocytopenia, thrombocytopathy, and exposure to anticoagulant or antiplatelet medication were excluded. Complete circumvallate placenta was defined as a placenta satisfying the following criteria: the chorionic plate, which is on the fetal side of the placenta, is smaller than the placental basal plate, which is located on the maternal side; the periphery is uncovered; and the fetal surface of such a placenta presents a central depression circumferentially surrounded by a thick, gray-white ring [6]. The circumvallate placenta group included women in whom circumvallate placenta was diagnosed by placental macroscopic examination performed after delivery by trained obstetricians and then confirmed by pathological examination. The control group included women who delivered a singleton after 22 gestational weeks during the same period, excluding those with circumvallate placenta.

Ninety-two women met the above criteria. Nine thousand fifty-seven women who had a morphologically normal placenta and delivered live singletons after 22 gestational weeks during the same period served as controls.

Subject background factors and pregnancy outcomes were compared between the two groups. The background factors were age, parity, and fertility treatment rate. Pregnancy outcomes were gestational week at delivery, vaginal bleeding during the 1st and 2nd trimesters, subchorionic hematoma, oligohydramnios, preterm PROM (pPROM), PCROM, placental abruption, and cesarean section rate. Neonatal outcomes, including birth weights, small-for-gestational age (SGA), neonatal death, neonatal intensive care unit (NICU) admission, and chronic lung disease (CLD), were also compared between 90 neonates in the circumvallate placenta group followed up for at least 4 weeks and those in the control group.

The circumvallate placenta group was divided into two subgroups: preterm (*n* = 59) and term (*n* = 33) delivery. We compared vaginal bleeding during the 1st and 2nd trimesters and PCROM between the two subgroups. In addition, we compared vaginal bleeding during the 2nd trimester + PCROM and vaginal bleeding during the 1st trimester + vaginal bleeding during the 2nd trimester + PCROM between the two subgroups. Similar comparisons were performed between the circumvallate placenta and control groups. Vaginal bleeding was defined as macroscopic blood detectable using a speculum. pPROM was defined as gross leakage of amniotic fluid before 37 gestational weeks with positive amniotic pH or insulin-like growth factor binding protein-1 (IGFBP-1) results. PCROM was defined as amniotic fluid leakage not grossly identifiable and difficult to differentiate from vaginal bleeding and diagnosed as rupture of membranes based on positive IGFBP-1 results [7]. Subchorionic hematoma was defined as a low echoic area around the gestational sac on ultrasonography during the 1st trimester. Oligohydramnios was defined as an amniotic fluid index <5 cm or amniotic pocket <2 cm. Placental abruption was diagnosed based on clinical findings, including vaginal bleeding, rigid abdomen, frequent uterine contractions, and late deceleration on fetal heart rate monitoring, necessitating emergency delivery [8]. The diagnosis was confirmed by the presence of a fresh retroplacental hematoma on postdelivery placental examination. SGA was defined as birth weight below the 10th percentile of the birth-weight-for-gestational-age reference curve [9]. CLD was defined as respiratory distress symptoms with an oxygen requirement starting in the neonatal period and persisting beyond age of 28 days due to pulmonary abnormalities other than congenital malformations.

Data are presented as medians or frequencies (%). Microsoft Excel and SPSS were used for statistical analyses. Fisher's exact tests were applied to compare categorical data between groups. The Mann-Whitney *U* test was used for continuous variables. Differences with a value of *P* < 0.05 were considered statistically significant. Moreover, linear regression and logistic regression were used to adjust for neonatal outcome, accounting for confounding variable (gestational week at delivery). And the results were expressed as odds ratios (OR) and 95% confidence intervals (CI).

## 3. Results

There are no significant differences in the maternal age, the primipara rate, or the fertility treatment rate between the circumvallate placenta and the control groups (Table 1).

Table 2 shows pregnancy outcomes of both groups. Gestational weeks at delivery were 32.8 in the circumvallate placenta group. The preterm delivery was significantly higher in the circumvallate placenta than in the control group. Particularly, the preterm delivery before 28 gestational weeks was significantly higher in the circumvallate placenta than in the control group. The incidences of vaginal bleeding during the 1st trimester and the 2nd trimester, subchorionic hematoma, oligohydramnios, pPROM, and placental abruption were also significantly higher in the circumvallate placenta than in the control group. The emergency cesarean section was significantly higher in the circumvallate placenta than in the control group.

Table 3 shows neonatal outcomes unadjusted and adjusted for the confounding variables (gestational week at delivery). Although the unadjusted analysis revealed an increased risk for UA pH < 7.10 and NICU admission in both the circumvallate placenta and the control groups, there were no differences in these variables between the two groups when the analysis was adjusted forgestational week at delivery.

Clinical symptoms were compared between the preterm (*n* = 59) and term (*n* = 33) delivery subgroups of the circumvallate placenta group (Table 4). Vaginal bleeding during the 1st and 2nd trimesters was significantly higher in the preterm than in the term delivery subgroup (the 1st trimester 42.3% versus 15.2%, *P* < 0.01; the 2nd trimester 52.5% versus 15.2%, *P* < 0.01). Vaginal bleeding during the 1st and 2nd trimesters with PCROM was 20.3% in the preterm delivery subgroup, while no cases in the term delivery subgroup experienced this complication.

As shown in Table 5, the incidences of both vaginal bleeding during the 2nd trimester with PCROM and vaginal bleeding during the 1st and 2nd trimesters with PCROM were significantly higher in the circumvallate placenta than in the control group (18.5% versus 0.1%, *P* < 0.01, and 13.0% versus 0.09%, *P* < 0.01, resp.). When vaginal bleeding during the 2nd trimester and PCROM were both used as predictions of circumvallate placenta, sensitivity was 28.8% and specificity was 99.9%.

## 4. Discussion

Vaginal bleeding during the 1st and 2nd trimesters, subchorionic hematoma, pPROM, oligohydramnios, preterm delivery, placental abruption, and emergency cesarean section were significantly higher in the circumvallate placenta than in the control group. Even when adjusted by gestational week at delivery, the incidences of SGA, neonatal death, and CLD were also significantly higher in the circumvallate placenta than in the control group, indicating poor neonatal outcomes. In women with circumvallate placenta who delivered before term especially, clinical symptoms including vaginal bleeding during the 1st and 2nd trimesters and PCROM were more common than in those delivering term babies. Notably, women with both vaginal bleeding during the 2nd trimester and PCROM accounted for 28.8% of the circumvallate placenta group delivering before term, as compared with 0.1% of controls. Thus, these two features might suggest circumvallate placenta during pregnancy.

The circumvallate placenta group shows poor pregnancy and delivery outcomes. Circumvallate placenta may lead to vaginal bleeding during the 2nd trimester, pPROM, and preterm delivery. A hematoma reportedly forms in the circumvallate placenta margin, causing ascending infection progressing to chorioamnionitis [10]. Suzuki [1] examined obstetrical outcomes in 139 circumvallate placenta cases and reported preterm delivery (22%), oligohydramnios (3.6%), placental abruption (5.0%), and emergency cesarean section (16%) incidences to be significantly higher in these women than in controls, observations consistent with our results.

SGA and CLD incidences were higher in the circumvallate placenta group than in controls. As to the association between circumvallate placenta and SGA, fetal growth restriction has been speculated to be caused by placental insufficiency due to a marginal infarct, hemorrhage, and hemosiderin deposition, in addition to a hypoplastic placenta [2, 11]. It has also been suggested that prolonged vaginal bleeding during pregnancy causes hemosiderin deposition in the placenta and chorion, resulting in diffuse chorioamniotic hemosiderosis, and that the fetus may swallow bloody amniotic fluid and thereby develop CLD [12, 13].

Women with circumvallate placenta frequently had clinical symptoms of vaginal bleeding during pregnancy and pPROM. Particularly, among those with circumvallate placenta who delivered before term, 28.8% had vaginal bleeding during the 2nd trimester and PCROM findings. Thus, these two features were supposed to be useful for antenatal prediction of circumvallate placenta. Bey et al. [14] say that when vaginal bleeding occurs in the 2nd trimester despite a normally implanted placenta, circumvallate placenta should be included in the differential diagnosis. Several studies have reported that circumvallate placenta can be diagnosed based on ultrasonographic abnormalities in placental appearance [2–5, 15]. McCarthy et al. [5] reported diagnostic criteria based on an irregular, uplifted placental edge (rounded placental margin) or a marginal shelf or rim (thin or sheet-like placental edge). Suzuki [2] described measurement of placental thickness (thickest part ≥3.0 cm) as being useful for circumvallate placenta screening. Arlicot et al. [15] recently reported that circumvallate placenta was diagnosed by 3-dimensional sonographic imaging showing a circumferential depression with a thick peripheral ring in the placenta. Harris et al. [3] found the accuracy of sonography for the diagnosis of complete circumvallate placenta to be 2% based on their criteria, concluding that it is difficult to diagnose this condition using sonography. In the present study, circumvallate placenta was suspected antenatally in only 4 (4.3%) of 92 women. In all four women, circumvallate placenta had been suspected based on a combination of characteristic clinical symptoms (i.e., oligohydramnios, vaginal bleeding during the 2nd trimester, and fetal growth restriction) and ultrasonographic findings (e.g., a thickened placenta and a hematoma at the placental margin). Circumvallate placenta was not suggested by ultrasonographic findings alone. Women with circumvallate placenta had characteristic clinical symptoms. When vaginal bleeding during the 2nd trimester and PCROM were both used as predictions of circumvallate placenta, sensitivity was 28.8% and specificity was 99.9%. In women with these two features, circumvallate placenta should be strongly suspected.

Our study has several limitations. First, the design was retrospective with a limited number of women. Second, the incidence of PCROM may have been underestimated because the IGFBP-1 test was not performed in all women in the circumvallate placenta and control groups. Third, case-control sampling can skew sensitivity, specificity, and calculations by producing an apparent disease prevalence that greatly overestimates the actual disease prevalence [16].

In summary, a circumvallate placenta was associated with higher incidences of not only maternal events, such as preterm delivery and placental abruption, but also fetal events, such as SGA, CLD, and neonatal death, indicating poor pregnancy and delivery outcomes. We demonstrated that predicting circumvallate placenta, though difficult, might be possible based on clinical symptoms. In women with both vaginal bleeding during the 2nd trimester and PCROM, circumvallate placenta should be suspected and their clinical courses should be carefully monitored.

## Figures and Tables

**Table 1:** Comparison of maternal characteristics between circumvallate placenta and control groups.

	Circumvallate placenta (*n* = 92)	Control (*n* = 9057)	*P* value
Maternal age (years)	31.9 (19–45)	31.6 (16–50)	0.76
Maternal age ≥35	25 (27.2%)	2691 (29.7%)	0.60
Primipara	46 (50%)	4579 (50.5%)	0.92
Spontaneous pregnancy	84 (91.3%)	8383 (92.5%)	0.65
Pregnancy by artificial insemination	2 (2.2%)	126 (1.4%)	0.53
Pregnancy by fertility drug	2 (2.2%)	194 (2.1%)	0.98
Pregnancy by in vitro fertilization	4 (4.3%)	355 (3.9%)	0.96

**Table 2:** Comparison of pregnancy outcomes between circumvallate placenta and control groups.

	Circumvallate placenta (*n* = 92)	Control (*n* = 9057)	*P* value
Gestational week at delivery (week)	32.8 (22.1–41.7)	38.2 (22.1–42.6)	<0.01
Extremely preterm delivery^*^	29 (31.5%)	214 (2.4%)	<0.01
Preterm delivery	59 (64.1%)	1069 (11.8%)	<0.01
VB during the 1st trimester	30 (32.6%)	85 (0.9%)	<0.01
VB during the 2nd trimester	36 (39.1%)	53 (0.6%)	<0.01
Subchorionic hematoma	14 (15.2%)	93 (1.0%)	<0.01
pPROM	36 (39.1%)	362 (4.0%)	<0.01
PCROM	19 (20.6%)	3 (0.03%)	<0.01
Oligohydramnios	22 (23.9%)	177 (2.0%)	<0.01
Placental abruption	10 (10.9%)	120 (1.3%)	<0.01
*Delivery modality *			
Normal vaginal delivery	42 (45.6%)	6397 (70.6%)	<0.01
Instrumental delivery	3 (3.2%)	419 (4.6%)	0.53
Elective cesarean section	4 (4.3%)	1062 (11.7%)	0.03
Emergency cesarean section	42 (45.6%)	1180 (13.0%)	<0.01

^*^Extremely preterm delivery: delivery at gestational age between 22 weeks 0 days and 27 weeks 6 days.

VB: vaginal bleeding; pPROM: preterm premature rupture of membranes; PCROM: premature chemical rupture of membranes; PIH: pregnancy-induced hypertension.

**Table 3:** Comparison of unadjusted and adjusted neonatal outcomes between circumvallate placenta and control groups.

	Control	Circumvallate placenta OR (95% CI)	*P* value
SGA			
Unadjusted	1	4.64 (3.02–7.12)	<0.01
Adjusted^*^	1	2.50 (1.58–3.95)	<0.01
UA pH < 7.10			
Unadjusted	1	4.65 (2.22–9.73)	<0.01
Adjusted^*^	1	0	0.81
NICU admission			
Unadjusted	1	8.68 (5.69–13.2)	<0.01
Adjusted^*^	1	0.50 (0.19–1.30)	0.16
Chronic lung disease			
Unadjusted	1	27.2 (15.9–46.6)	<0.01
Adjusted^*^	1	2.92 (1.30–6.56)	<0.01
Neonatal death			
Unadjusted	1	32.6 (14.4–74.0)	<0.01
Adjusted^*^	1	2.70 (1.03–7.10)	<0.05

SGA: small-for-gestational age; UA pH: umbilical artery pH; NICU: neonatal intensive care unit; SE: standard error; OR: odds ratio.

^*^Adjusted by gestational weeks at delivery.

**Table 4:** Comparison of pregnancy outcomes between preterm and term delivery subgroups of circumvallate placenta group.

	Preterm delivery (*n* = 59)	Term delivery (*n* = 33)	*P* value
VB during the 1st trimester	25 (42.3%)	5 (15.2%)	0.010
VB during the 2nd trimester	31 (52.5%)	5 (15.2%)	<0.01
Subchorionic hematoma	11 (18.6%)	3 (9.1%)	0.36
PROM	36 (61.0%)	5 (15.2%)	<0.01
pPROM	36 (61.0%)	0 (0%)	<0.01
PCROM	19 (32.2%)	0 (0%)	<0.01
VB during the 2nd trimester + PCROM	17 (28.8%)	0 (0%)	<0.01
VB during the 1st and 2nd trimesters + PCROM	12 (20.3%)	0 (0%)	<0.01

VB: vaginal bleeding; PROM: premature rupture of membranes; pPROM: preterm premature rupture of membranes; PCROM: premature chemical rupture of membranes.

**Table 5:** Comparison of clinical symptom rates between circumvallate placenta and control groups.

	Circumvallate placenta (*n* = 92)	Control (*n* = 9057)	*P* value
VB during the 2nd trimester + PCROM	17 (18.5%)	9 (0.1%)	<0.01
VB during the 1st and 2nd trimesters + PCROM	12 (13.0%)	8 (0.09%)	<0.01

VB: vaginal bleeding; PCROM: premature chemical rupture of membranes.
